# Valorization of Artichoke Bracts as a Simultaneous Fat and Wheat Flour Substitute in Cookies

**DOI:** 10.1111/1750-3841.71357

**Published:** 2026-08-10

**Authors:** Ceyda Dadalı, Yağmur Özcan

**Affiliations:** ^1^ Department of Food Engineering, Faculty of Engineering Ege University İzmir Türkiye

**Keywords:** artichoke bracts, cookies, *Cynara scolymus* L., fat substitute, sustainability, wheat flour substitute

## Abstract

The valorization of food processing byproducts has become increasingly important for sustainable food production. Artichoke processing generates substantial amounts of waste, particularly in the form of bracts, which are rich in bioactive compounds and dietary fiber. This study investigated the potential of artichoke bracts as a simultaneous substitute for fat and wheat flour in cookie formulations and optimized the substitution levels using response surface methodology (RSM). The optimal formulation contained 14.26% artichoke bracts as a fat substitute and 23.74% as a wheat flour substitute. The optimized cookies exhibited significantly higher moisture content, hardness, fracturability, total phenolic content, and antioxidant activity (*p* < 0.05). The total dietary fiber content also increased significantly, reaching 10.14 g/100 g, allowing the product to be classified as a high‐fiber food. In contrast, baking loss, spread ratio, and color parameters (L*, a*, b*) decreased with increasing substitution levels. Sensory evaluation demonstrated improvements in color, texture, flavor, and overall acceptability under optimal conditions. The findings indicate that artichoke bracts can be effectively utilized as partial substitutes for fat and wheat flour, enhancing the nutritional quality of cookies while maintaining sensory properties. This approach provides a promising strategy for food waste valorization and the development of functional bakery products.

**Practical Applications:**

Artichoke bracts can be used as substitutes for fat and wheat flour in cookie formulations, enabling the production of fiber‐rich and antioxidant‐enhanced bakery products without compromising sensory quality. This approach offers the food industry a practical solution for reducing processing waste while supporting the development of sustainable and value‐added functional foods.

## 1 Introduction

Food byproducts refer to organic materials produced throughout different stages of food production and processing, ranging from primary agricultural production and harvesting to postharvest operations and industrial processing activities (Melini et al. 2020). These materials are typically not destined for direct dietary use and are often treated as waste. The management and disposal of these residues generate financial costs and intensify environmental impacts, particularly through greenhouse gas emissions and the growing demand for landfill space (Vilas‐Franquesa et al. 2024).

Artichoke (*Cynara scolymus* L.) is cultivated and consumed globally, largely owing to its distinctive taste and valuable nutritional composition. Artichoke has been recognized for its diverse biological activities, including antioxidant, hypocholesterolemic, hepatoprotective, antimicrobial, antitumor, and anti‐inflammatory effects. The observed functional effects are primarily linked to the abundance of phenolic constituents in artichoke, notably mono‐ and dicaffeoylquinic acids, including chlorogenic acid and cynarin, as well as flavonoids like luteolin and apigenin and their respective glycoside and rutinoside forms (Cannas et al. 2024). Furthermore, artichoke provides a substantial amount of dietary fiber, predominantly in the form of insoluble fiber, which constitutes about 75% of the total. The insoluble fraction primarily contains cellulose, hemicellulose, and lignin, while inulin and pectin constitute the major components of the soluble dietary fiber fraction (Ayuso et al. 2024). According to data from FAOSTAT (FAO 2025), global artichoke production reached 1,816,169 tons in 2024. A substantial proportion of the biomass obtained from artichoke processing, approximately 80%–85%, is represented by processing byproducts (De Falco et al. 2022). Bracts, leaves, and stems are the main byproducts obtained from industrial artichoke processing, and, like the edible portions, these fractions are rich in bioactive antioxidant constituents. These byproducts are typically transformed into powdered form and subsequently incorporated into bakery products (Cannas et al. 2024).

Cookies represent one of the most frequently consumed products within the bakery sector, valued for their distinctive sensory properties, particularly their flavor and texture. Fat and flour are the two main components in cookie formulations, and they have an influence on the structure and sensory properties of cookies (Meral et al. 2024). Growing global concern about human health has intensified interest in dietary patterns and their long‐term effects (Sudarshan and Sharan 2023). High intake of fat‐rich and high‐calorie foods has been linked to excessive energy consumption and an increased risk of obesity. As awareness of the adverse consequences of overconsumption continues to rise, the food industry has placed growing emphasis on the development of reduced‐fat products (Fadillah et al. 2024). High fat and wheat flour content in cookies increases caloric density and potential health concerns, promoting the investigation of substitutes that can reduce energy content while maintaining quality attributes (Yalcin et al. 2023). The valorization of byproducts as fat and flour substitutes in food formulation has gained attention to improve nutritional quality, promote sustainability, and create added value (Gómez and Martinez 2018; Plazzotta et al. 2023).

The potential use of artichoke bracts as functional ingredients in food matrices such as rusks, bread, cookies, cakes, muffins, and pasta has been explored in previous studies (Canale et al. 2023; Dadalı 2023; Proetto et al. 2024; Bavaro, De Bellis, Linsalata, et al. 2025; Dadalı et al. 2025; Gulsunoglu‐Konuskan et al. 2025; Melini et al. 2025). Based on the available literature, the combined application of artichoke bracts as dual fat and wheat flour substitutes in cookies has not yet been investigated. The objective of the present study is to assess artichoke bracts as a fat and wheat flour substitute and to optimize their incorporation levels to produce cookies with improved nutritional composition and enhanced quality characteristics.

## 2 Materials and Methods

Margarine, wheat flour, sucrose, eggs, and baking powder were sourced from a market, while artichokes were obtained from a producer in Türkiye. The chemicals were supplied by Merck (Germany).

### 2.1 Experimental Design

Response surface methodology (RSM) was applied to determine the optimum levels of artichoke bracts as substitutes for fat and wheat flour in cookie formulations. The experimental runs used to model the relationships between the independent variables and the measured responses were structured according to a Face Centered Central Composite Design (FCCCD). All statistical evaluations and optimization analyses were carried out by Design‐Expert software (v. 13.0). The study considered two independent numerical variables, fat substitute level and wheat flour substitute level, both ranging from 0% to 30%. To determine the optimum substitution levels in cookie formulations, multiple response parameters were considered, including moisture content, baking loss, spread ratio, hardness, fracturability, L*, a*, and b* color values, total phenolic content, antioxidant activity, and sensory attributes. Predictive models for each response were established through regression analysis of experimental findings (Equation 1). Within the polynomial expression, *R* indicates the calculated response, *β*_0_ refers to the intercept, and *β_i_*, *β_i__i_*, and *β_i__j_* correspond to the linear, second‐order, and interaction parameters, respectively:

(1) $$R=\beta_{0}+\sum \beta_{i}X_{i}+\sum \beta_{ii}X_{ii}^{2}+\sum \beta_{ij}X_{i}X_{j}.$$

### 2.2 Production of Artichoke Bracts

Artichokes were obtained from local producers, and the bracts were separated and washed within 24 h of procurement. The cleaned bracts were subjected to drying using a laboratory‐scale tray dryer (Eksis, Türkiye) set at 50°C with an air velocity of 1.2 m/s. The process was maintained until samples reached a moisture level under 10% (Sałata et al. 2022). Following drying, the bracts were pulverized using a laboratory‐scale grinder (Waring, USA) and sieved to a 283 µm particle size, and the resulting powder was incorporated into cookie dough formulations.

### 2.3 Cookie Preparation

The control cookie formulation was prepared by blending margarine (50 g) and sucrose (24 g) in a mixer at medium speed for 4 min. Subsequently, egg (7.01 g), baking powder (2.00 g), and wheat flour (100.00 g) were incorporated, and the mixture was kneaded for 6 min until a uniform structure was achieved. According to design, artichoke bracts were incorporated into the cookie formulation as a fat substitute (0%–30%) and as a wheat flour substitute (0%–30%) (Table 1). The dough was rolled out to obtain a consistent thickness of 1.5 cm and cut into circular pieces (3 cm diameter) using a round cutter. The prepared samples were baked at 190°C for 20 min in a preheated oven (Arçelik, Türkiye), and subsequently cooled at room temperature for 1 h prior to analysis.

**TABLE 1:** Design of experiments and response variables based on FCCCD.

Run	Fat substitute (%)	Wheat flour substitute (%)	Moisture (%)	Baking loss (%)	Spread ratio	Hardness (N)	Fracturability (mm)	L*	a*	b*	Total phenolic compounds (mg GAE/g)	Antioxidant activity (µmol TE/g)	Color	Texture	Flavor	Overall acceptability
1	15	15	12.54	12.66	1.80	40.94	50.65	63.58	4.23	25.14	0.83	2.25	7.59	7.45	8.25	8.19
2	30	15	12.71	11.43	1.77	38.99	52.46	55.31	3.56	22.72	0.98	3.17	7.93	7.31	7.92	7.80
3	15	15	12.42	12.60	1.83	41.25	50.98	62.75	4.45	26.03	0.87	2.18	7.45	7.42	8.23	8.23
4	15	30	12.96	11.12	1.69	44.18	53.29	53.79	3.29	21.31	1.14	4.32	8.26	6.80	7.95	8.15
5	15	15	12.49	12.76	1.83	41.89	51.25	63.85	4.39	25.95	0.85	2.31	7.62	7.49	8.19	8.26
6	0	15	11.73	12.90	2.07	34.95	49.19	68.13	4.72	29.25	0.73	2.13	7.21	7.42	7.87	7.81
7	30	0	12.09	12.27	1.93	35.07	49.74	70.28	4.69	28.67	0.68	2.01	7.35	7.36	7.69	7.55
8	0	0	9.40	14.57	2.56	29.03	47.43	82.15	7.36	35.38	0.22	0.58	6.15	6.98	7.55	7.03
9	15	15	12.39	12.81	1.85	42.03	51.29	62.48	4.19	26.54	0.88	2.27	7.40	7.42	8.27	8.24
10	15	15	12.58	12.79	1.84	42.19	50.85	63.56	4.25	25.85	0.83	2.16	7.52	7.47	8.29	8.21
11	0	30	12.68	11.68	1.73	36.98	51.89	60.89	3.98	23.05	0.96	4.03	8.14	7.22	7.78	8.02
12	15	0	10.98	13.98	2.21	34.75	48.01	78.56	6.29	31.09	0.43	1.20	6.83	7.25	7.85	7.73
13	30	30	13.09	10.24	1.61	45.01	55.29	44.68	2.95	18.56	1.27	5.03	8.49	6.52	7.59	7.26

### 2.4 Composition Analysis

The compositional analysis of the cookie samples included the determination of moisture, ash, fat, protein, and dietary fiber contents in accordance with the respective AOAC official methods (925.10, 923.03, 945.16, 968.06, and 991.41) as described by AOAC (2007).

### 2.5 Baking Loss

To determine baking loss, the cookie dough was weighed prior to baking, and the baked cookies were weighed after cooling at room temperature for 1 h. Baking loss (%) was determined by calculating the mass reduction during baking relative to the initial dough weight. The difference between the pre‐baking and post‐baking weights was divided by the initial dough mass and expressed as a percentage.

### 2.6 Spread Ratio

The spread ratio was assessed by measuring the width and thickness of baked samples with a digital caliper and calculating their ratio in accordance with AACC (2000). Cookie thickness was measured by stacking four samples vertically and measuring their cumulative height. The order of the cookies was subsequently altered, and the measurement was performed again to obtain a representative value. The average of the two measurements was calculated and divided by 4 to obtain the mean cookie thickness. For each formulation, four cookies were evaluated, and diameter values were obtained from two perpendicular measurements for each sample to calculate a mean diameter. The spread ratio was calculated by relating this value to the corresponding average thickness.

### 2.7 Texture Analysis

The textural characteristics of the samples were determined using a TA.XT Plus texture analyzer (Stable Micro Systems Ltd., Godalming, UK) coupled with a 5‐kg load cell and a three‐point bending rig (HDP/3 PB).

Textural analysis was conducted with a TA.XT Plus texture analyzer (Stable Micro Systems Ltd.) fitted with a 5‐kg load cell and a three‐point bending rig (HDP/3 PB). Cookie hardness and fracturability were measured through a three‐point bending test conducted at 1.0 mm/s (pretest) and 3.0 mm/s (test), with a deformation distance of 5.0 mm, as outlined by Kim et al. (2019).

### 2.8 Color Analysis

The surface color of the baked cookies was measured on the upper side using a CR‐300 colorimeter (Konica Minolta, Japan). Color values were expressed as L* (lightness), a* (redness/greenness), and b* (yellowness/blueness).

### 2.9 Total Phenolic Content

For phenolic extraction, 2.5 g of powdered cookie was treated with 20 mL of 80% methanol (v/v) and maintained at 50°C for 90 min under continuous shaking (Memmert, Germany). Following centrifugation at 4500 rpm for 5 min, the extract was filtered, and its volume was adjusted to 25 mL as outlined by Garcia‐Salas et al. (2010). The total phenolic content was analyzed using a modified Folin–Ciocalteu assay adapted from Heimler et al. (2006). The extract (50 µL) was diluted with 3 mL distilled water, after which 250 µL of Folin–Ciocalteu reagent was introduced. Then, 750 µL of 7% (w/v) Na_2_CO_3_ was added, and the mixture was allowed to stand at ambient temperature for 8 min. It was subsequently diluted with 950 µL of distilled water and kept in darkness for 2 h. Absorbance readings were taken at 765 nm with a UV–Vis spectrophotometer (Agilent Cary 60, USA). The concentration of total phenolics was calculated based on a gallic acid calibration curve and reported as mg GAE/g sample.

### 2.10 Antioxidant Activity

A modified DPPH radical scavenging assay, based on the methods of Tomaino et al. (2010) and Rapisarda et al. (2008), was used to determine antioxidant activity. The extracts obtained for total phenolic analysis were used in the assay. The assay was performed by mixing 25–75 µL of the extract with 1950 µL of 100 µM DPPH solution, after which the mixtures were incubated at room temperature for 20 min prior to measuring absorbance at 517 nm with a spectrophotometer. Antioxidant capacity was determined from radical scavenging activity (%) and expressed as µmol Trolox equivalents (TE)/g sample.

### 2.11 Sensory Analysis

Sensory attributes were assessed using a 9‐point hedonic scale, where scores ranged from 1 (*dislike extremely*) to 9 (*like extremely*). The evaluation involved 63 panelists aged between 18 and 57 years. Each cookie sample was labeled with a randomly generated three‐digit code prior to evaluation. Participants assessed the products for color, texture, flavor, and overall acceptability in accordance with the approach described by Altuğ Onoğur and Elmacı (2015). This study received ethical approval from the Scientific Research Ethics Committee of Ege University under approval number 2438.

## 3 Results and Discussion

### 3.1 Model Descriptions

The impact of incorporating artichoke bracts as a substitute for fat and wheat flour in cookies was assessed through RSM using an FCCCD. The results of the analyses, conducted according to a 13‐trial experimental design, are given in Table 1. ANOVA results demonstrated that all models were statistically significant (*p* < 0.01), indicating their adequacy for predicting the effects of artichoke bract substitution for fat and wheat flour on the responses (Table 2). The ANOVA analysis revealed a nonsignificant lack‐of‐fit (*p* > 0.05), suggesting that the developed models were appropriate. Moreover, *R* ^2^ and adjusted *R* ^2^ values greater than 0.9571 indicated a high level of consistency between the observed data and the model predictions. Furthermore, Adeq Precision values, which were considerably higher than the threshold value of 4, indicated an adequate signal‐to‐noise ratio. The coefficient of variation (CV), used as a measure of experimental precision, was below 5% for all responses, indicating excellent reproducibility and reliability. Moisture content, baking loss, spread ratio, hardness, L*, a*, b*, total phenolic compounds, antioxidant activity, color, texture, flavor, and overall acceptability, selected as responses, were explained by a quadratic model, while fracturability was explained by a linear model (Table 3).

**TABLE 2:** ANOVA results for the response variables.

*R*_1_: Moisture			*R*_2_: Baking loss		
*R* ^2^: 0.9877, Adj‐*R* ^2^: 0.9782, %CV: 1.2100, Adeq Precision: 35.6358			*R* ^2^: 0.9894, Adj‐*R* ^2^: 0.9819, %CV: 1.2400, Adeq Precision: 41.3265		
	*F*‐value	*p*‐value		*F*‐value	*p*‐value
Model	108.74	0.00	Model	131.15	0.00
*X*_1_	129.17	0.00	*X*_1_	190.2	0.00
*X*_2_	304.09	0.00	*X*_2_	424.13	0.00
*X*_1_ *X*_2_	60.51	0.00	*X*_1_ *X*_2_	7.77	0.03
*X*_1_^2^	5.75	0.04	*X*_1_^2^	24.88	0.00
*X*_2_^2^	27.37	0.00	*X*_2_^2^	0.71	0.43
Lack of fit	6.58	0.05	Lack of fit	5.49	0.07

*R*_3_: Spread ratio			*R*_4_: Hardness		
*R* ^2^: 0.9882, Adj‐*R* ^2^: 0.9798, %CV: 1.8800, Adeq Precision: 35.6358			*R* ^2^: 0.9750, Adj‐*R* ^2^: 0.9571, %CV: 2.4400, Adeq Precision: 23.9489		
	*F*‐value	*p*‐value		*F*‐value	*p*‐value
Model	117.33	0.00	Model	54.51	0.00
*X*_1_	143.28	0.00	*X*_1_	60.43	0.00
*X*_2_	362.44	0.00	*X*_2_	137.53	0.00
*X*_1_ *X*_2_	50.7	0.00	*X*_1_ *X*_2_	1.09	0.33
*X*_1_^2^	5.42	0.06	*X*_1_^2^	45.17	0.00
*X*_2_^2^	13.84	0.01	*X*_2_^2^	5.58	0.05
Lack of Fit	7.22	0.05	Lack of Fit	5.96	0.06

*R*_5_: Fracturability			*R*_6_: L*		
*R* ^2^: 0.9818, Adj‐*R* ^2^: 0.9781, %CV: 0.6122, Adeq Precision: 53.9902			*R* ^2^: 0.9978, Adj‐*R* ^2^: 0.9963, %CV: 0.9394, Adeq Precision: 92.0565		
	*F*‐value	*p*‐value		*F*‐value	*p*‐value
Model	269.31	0.00	Model	646.07	0.00
*X*_1_	138.14	0.00	*X*_1_	775.03	0.00
*X*_2_	400.47	0.00	*X*_2_	2377.18	0.00
Lack of fit	1.56	0.35	*X*_1_ *X*_2_	13.09	0.01
			*X*_1_^2^	19.58	0.01
			*X*_2_^2^	62.72	0.00
			Lack of Fit	1.05	0.46

*R*_7_: a*			*R*_8_: b*		
*R* ^2^: 0.9831, Adj‐*R* ^2^: 0.9710, %CV: 4.4800, Adeq Precision: 31.6879			*R* ^2^: 0.9913, Adj‐*R* ^2^: 0.9850, %CV: 2.0500, Adeq Precision: 45.8437		
	*F*‐value	*p*‐value		*F*‐value	*p*‐value
Model	81.45	0.00	Model	159.04	0.00
*X*_1_	97.46	0.00	*X*_1_	183.32	0.00
*X*_2_	272.05	0.00	*X*_2_	605.39	0.00
*X*_1_ *X*_2_	16.65	0.01	*X*_1_ *X*_2_	4.31	0.08
*X*_1_^2^	0.76	0.41	*X*_1_^2^	0.21	0.66
*X*_2_^2^	20.27	0.00	*X*_2_^2^	1.26	0.29
Lack of Fit	6.19	0.06	Lack of Fit	1.31	0.39

*R*_9_: Total phenolic compounds			*R*_10_: Antioxidant activity		
*R* ^2^: 0.9901, Adj‐*R* ^2^: 0.9831, %CV: 4.3300, Adeq Precision: 42.2233			*R* ^2^: 0.9958, Adj‐*R* ^2^: 0.9928, %CV: 4.0900, Adeq Precision: 60.6185		
	*F*‐value	*p*‐value		*F*‐value	*p*‐value
Model	140.5	0.00	Model	330.18	0.00
*X*_1_	137.14	0.00	*X*_1_	179.59	0.00
*X*_2_	548.56	0.00	*X*_2_	1371.71	0.00
*X*_1_ *X*_2_	4.45	0.07	*X*_1_ *X*_2_	4.14	0.08
*X*_1_^2^	0.0003	0.99	*X*_1_^2^	20.61	0.00
*X*_2_^2^	10.6	0.01	*X*_2_^2^	39.31	0.00
Lack of Fit	4.34	0.10	Lack of Fit	5.3	0.07

*R*_11_: Color			*R*_12_: Texture		
*R* ^2^: 0.9903, Adj‐*R* ^2^: 0.9833, %CV: 1.0600, Adeq Precision: 42.1476			*R* ^2^: 0.9843, Adj‐*R* ^2^: 0.9731, %CV: 0.6716, Adeq Precision: 29.5094		
	*F*‐value	*p*‐value		*F*‐value	*p*‐value
Model	142.57	0.00	Model	87.95	0.00
*X*_1_	135.85	0.00	*X*_1_	13.04	0.0
*X*_2_	548.19	0.00	*X*_2_	77.73	0.00
*X*_1_ *X*_2_	28.57	0.00	*X*_1_ *X*_2_	123.36	0.00
*X*_1_^2^	0.2095	0.66	*X*_1_^2^	2.51	0.16
*X*_2_^2^	0.0042	0.95	*X*_2_^2^	174.43	0.00
Lack of Fit	0.396	0.76	Lack of Fit	4.47	0.09

*R*_13_: Flavor			*R*_14_: Overall acceptability		
*R* ^2^: 0.9816, Adj‐*R* ^2^: 0.9684, %CV: 0.5932, Adeq Precision: 22.3909			*R* ^2^: 0.9945, Adj‐*R* ^2^: 0.9907, %CV: 0.4936, Adeq Precision: 46.3094		
	*F*‐value	*p*‐value		*F*‐value	*p*‐value
Model	74.65	0.00	Model	255.34	0.00
*X*_1_	0	1.00	*X*_1_	6.88	0.03
*X*_2_	3.96	0.09	*X*_2_	138.09	0.00
*X*_1_ *X*_2_	12.22	0.01	*X*_1_ *X*_2_	270.55	0.00
*X*_1_^2^	112.37	0.00	*X*_1_^2^	364.62	0.00
*X*_2_^2^	108.67	0.00	*X*_2_^2^	177.66	0.00
Lack of Fit	2.18	0.23	Lack of Fit	3.51	0.13

*Note: X*_1_ = fat substitute; *X*_2_ = wheat flour substitute.

**TABLE 3:** Model equations of responses.

Response	Equation
Moisture content	12.4700 + 0.6800 *X*_1_ + 1.04 *X*_2_ − 0.5700 *X*_1_ *X*_2_ − 0.2114 *X*_1_^2^ − 0.4614 *X*_2_^2^
Baking loss	12.700 − 0.8686 *X*_1_ − 1.30 *X*_2_ + 0.2150 *X*_1_ *X*_2_ − 0.4629 *X*_1_^2^ − 0.0779 *X*_2_^2^
Spread ratio	1.8400 − 0.1750 *X*_1_ − 0.2783 *X*_2_ + 0.1275 *X*_1_ *X*_2_ + 0.0502 *X*_1_^2^ + 0.0802 *X*_2_^2^
Hardness	41.4200 + 3.0200 *X*_1_ + 4.5500 *X*_2_ + 0.4975 *X*_1_ *X*_2_ − 3.8500 *X*_1_^2^ − 1.3500 *X*_2_^2^
Fracturability	50.9500 + 1.5000 *X*_1_ + 2.5500 *X*_2_
L*	63.2600 − 6.8200 *X*_1_ − 11.9400 *X*_2_ − 1.0800 *X*_1_ *X*_2_ − 1.6000 *X*_1_^2^ + 2.8600 *X*_2_^2^
a*	4.2900 − 0.8100 *X*_1_ − 1.3500 *X*_2_ − 0.4100 *X*_1_ *X*_2_ − 0.1055 *X*_1_^2^ + 0.5445 *X*_2_^2^
b*	25.8800 − 2.9600 *X*_1_ − 5.3700 *X*_2_ + 0.5550 *X*_1_ *X*_2_ + 0.1467 *X*_1_^2^ + 0.3617 *X*_2_^2^
Total phenolic compounds	0.8528 − 0.1700 *X*_1_ − 0.3400 *X*_2_ + 0 *X*_1_ *X*_2_ + 0.1467 *X*_1_^2^ + 0.3617 *X*_2_^2^
Antioxidant activity	2.2700 + 0.5783 *X*_1_ + 1.6000 *X*_2_ − 0.1075 *X*_1_ *X*_2_ + 0.2888 *X*_1_^2^ + 0.3988 *X*_2_^2^
Color	7.5300 + 0.3783 *X*_1_ + 0.7600 *X*_2_ − 0.2125 *X*_1_ *X*_2_ + 0.0219 *X*_1_^2^ − 0.0031 *X*_2_^2^
Texture	7.4400 − 0.0717 *X*_1_ − 0.1750 *X*_2_ − 0.2700 *X*_1_ *X*_2_ − 0.0464 *X*_1_^2^ − 0.3864 *X*_2_^2^
Flavor	8.2300 + 0.0000 *X*_1_ + 0.0383 *X*_2_ − 0.0825 *X*_1_ *X*_2_ − 0.3010 *X*_1_^2^ − 0.2960 *X*_2_^2^
Overall acceptability	8.2300 − 0.0417 *X*_1_ + 0.1867 *X*_2_ − 0.3200 *X*_1_ *X*_2_ − 0.4471 *X*_1_^2^ − 0.3121 *X*_2_^2^

*Note: X*_1_ = fat substitute; *X*_2_ = wheat flour substitute.

### 3.2 Effect of Using Artichoke Bracts on Physical Properties of Cookies

**FIGURE 1:**
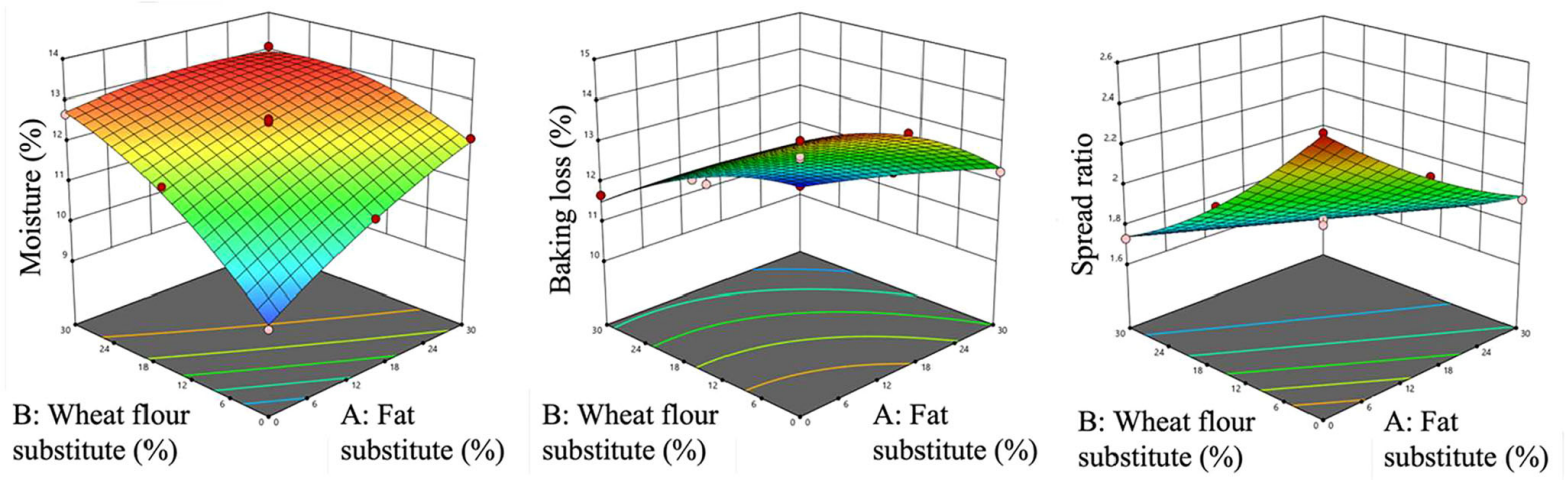
The calculated effect of fat and wheat flour substitution on the physical properties of cookies.

The substitution of fat and wheat flour with artichoke bracts significantly affected moisture content through linear and quadratic effects (*p* < 0.05), along with a significant interaction between the two factors (Table 2). In both cases, higher levels of artichoke bract incorporation led to increased moisture content in cookies (Figure 1). The control formulation (Exp. 8), without artichoke bract substitution for fat or wheat flour, exhibited the lowest moisture content (9.40%) (Table 1). Conversely, the highest value (13.09%) was obtained in Exp. 13, where 30% artichoke bracts were used as both fat and wheat flour substitutes. Artichoke bracts are known to exhibit high water‐holding capacity, primarily due to their elevated fiber content and the presence of hydrophilic compounds. Since high water‐holding capacity increases the dough's ability to retain water, moisture content increased as the ratio of artichoke bracts increased (Amoah et al. 2020; Bavaro, De Bellis, Montemurro, et al. 2025). A negative relationship was observed between moisture content and baking loss, with the lowest moisture sample (Exp. 8) corresponding to the highest baking loss value (14.57%). Conversely, the lowest baking loss (10.24%) was found in the cookie with the highest moisture content (Exp. 13). Similar trends have been reported in the literature, where Cannas et al. (2024) and Bavaro, De Bellis, Montemurro, et al. (2025) documented increased moisture levels in breadsticks and bread enriched with artichoke byproducts and bracts, respectively, compared to control formulations. Cookie spread ratios were observed within the range of 1.61–2.56 (Table 1). While fat substitution exhibited a linear effect on the spread ratio, wheat flour substitution showed both linear and quadratic effects (*p* < 0.05). An interaction effect between fat substitution and wheat flour substitution was detected regarding the spread ratio (*p* < 0.05) (Table 2). Spread ratio showed a decreasing trend with increasing substitution levels of fat and wheat flour (Figure 1). The lowest value (1.61) was recorded in the formulation containing 30% substitution for both fat and wheat flour, whereas the control sample exhibited the highest spread ratio (2.56). According to Ebere et al. (2015), an increase in substitution level in apple–cashew fiber cookies resulted in a reduction in spread ratio. This effect was explained by the presence of gluten in wheat flour, which is responsible for dough elasticity and spreading behavior during baking; consequently, the control sample exhibited a higher spread ratio. A decreasing trend in spread ratio with increasing replacement level was also reported by Mudgil et al. (2017) in cookies fortified with partially hydrolyzed guar gum as a soluble fiber. In parallel with these studies, it was also determined that as the substitution rate increased, the spread ratio decreased in cookies containing jujube powder, which has a high fiber content (Urooj et al. 2026).

### 3.3 Effect of Using Artichoke Bracts on Textural Properties of Cookies

**FIGURE 2:**
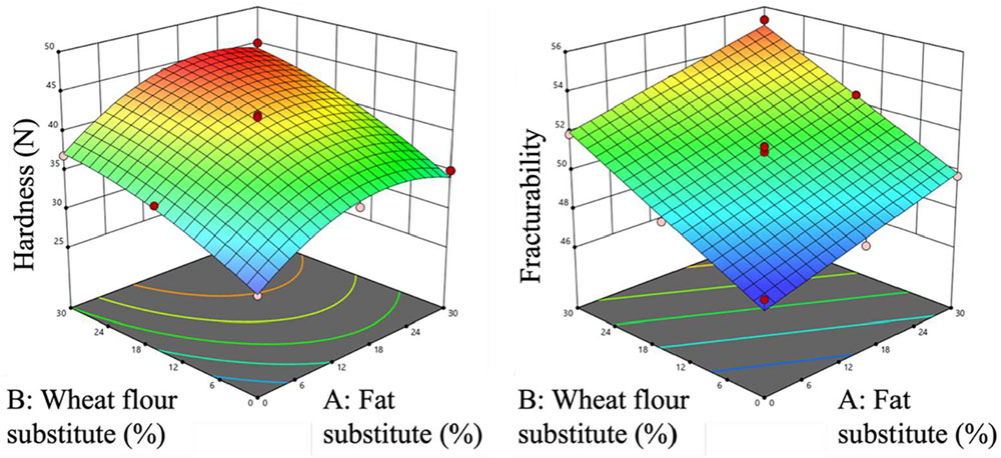
The calculated effect of fat and wheat flour substitution on the textural properties of cookies.

The influence of artichoke bracts as a fat and wheat flour substitute on cookie hardness and fracturability was investigated in this study. Significant linear and quadratic effects of fat substitution, along with a significant linear effect of wheat flour substitution, were observed for hardness (*p* < 0.05), while the interaction effect was not significant (*p* > 0.05) (Table 2). Overall, hardness increased with rising levels of substitution (Figure 2). The control sample exhibited the lowest hardness (29.03 N), whereas the cookie with 30% fat and 30% wheat flour substitution showed the highest value (45.01 N). Cookie fracturability showed a pattern similar to that observed for hardness. It was found that as the substitution ratio increased, fracturability values also increased (Figure 2). In breads enriched with artichoke bracts, it was determined that hardness increased as the addition ratio of artichoke bracts increased (Bavaro, De Bellis, Montemurro, et al. 2025). In a study conducted by Barakat et al. (2024), it was observed that adding Jerusalem artichoke flour to gluten‐free biscuits increased hardness and fracturability. Similarly, it was determined that adding celery root powder, which is high in fiber, to cookies increased their hardness and fracturability values. The observed increases in hardness and fracturability were associated with the use of fiber‐rich, gluten‐free ingredients in the cookie formulation. This phenomenon has been explained by the fact that polysaccharides interfere with the interactions between gluten proteins, thereby limiting the formation of a continuous and elastic gluten matrix. Additionally, the strong water‐binding ability of fiber components reduced water availability for gluten hydration, which hindered gluten network formation and resulted in a denser and harder structure. A heterogeneous and discontinuous structure of fiber particles within the dough matrix has led to easier breakage of the product under applied force, thereby increasing its fracturability values (Nićetin et al. 2024).

### 3.4 Effect of Using Artichoke Bracts on Color Properties of Cookies

**FIGURE 3:**
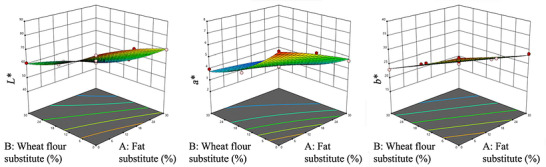
The calculated effect of fat and wheat flour substitution on the color properties of cookies.

Significant linear and quadratic effects of fat and wheat flour substitution levels on L* values were observed (*p* < 0.05) (Table 2). The incorporation of artichoke bracts as a substitute for fat and wheat flour led to a reduction in L* values (Figure 3), with the highest value recorded in the control sample (82.15; Exp. 8) and the lowest in Exp. 13 (44.68). This could be explained by the naturally dark color of artichoke bract powder and the intensification of Maillard browning reactions on the cookie surface during baking. Regarding a* values, an increasing trend was observed with higher substitution levels (Figure 3). Fat substitution showed a significant linear effect on a*, whereas wheat flour substitution exhibited both linear and quadratic effects (*p* < 0.05). In addition, the interaction between the two substitution factors significantly affected both L* and a* values (*p* < 0.05). This increase was likely associated with the formation of brown‐red pigments through Maillard reactions and the oxidation of phenolic compounds present in artichoke bracts. For b* values, only linear effects of fat and wheat flour substitution were significant (*p* < 0.05), and a decreasing trend was observed as the substitution level increased (Figure 3). This decrease may have resulted from the partial replacement of wheat flour, which naturally contains yellow carotenoid pigments, together with the darker color imparted by artichoke bracts. Cookie b* values varied between 18.56 and 35.38 (Table 1), in line with the results reported in the literature by Cansev et al. (2024) and Cannas et al. (2024). In the studies conducted, it was observed that as the level of artichoke flour added to the cookies and breadsticks increased, the L*, a*, and b* values decreased (Cansev et al. 2024; Cannas et al. 2024).

### 3.5 Effect of Using Artichoke Bracts on Total Phenolic Compounds and Antioxidant Activity of Cookies

**FIGURE 4:**
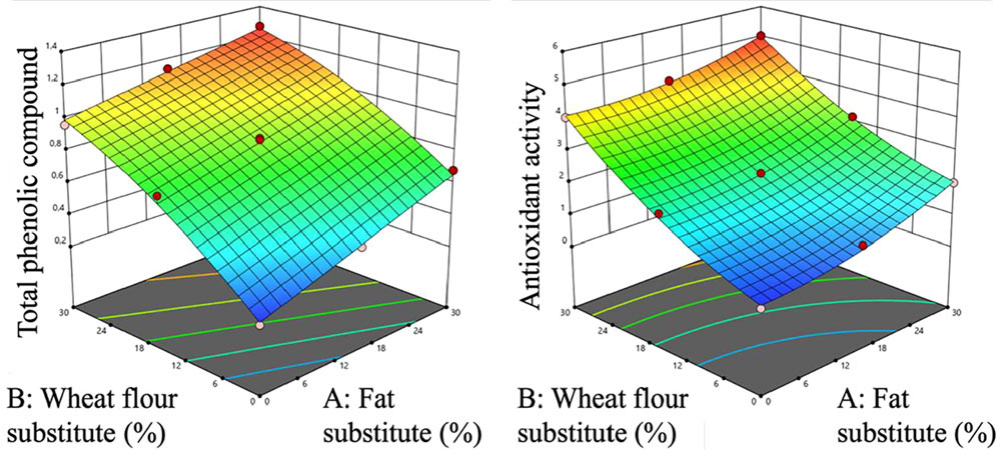
The calculated effect of fat and wheat flour substitution on total phenolic compounds and antioxidant activity of cookies.

A significant linear effect on total phenolic content was observed for fat substitution, while wheat flour substitution showed both linear and quadratic effects (*p* < 0.05) (Table 2). Total phenolic content increased progressively with higher levels of artichoke bract incorporation (Figure 4), rising from 0.22 mg GAE/g in the control to 1.27 mg GAE/g at 30% substitution of both fat and wheat flour. Antioxidant activity values ranged between 0.58 and 5.03 µmol TE/g (Table 1). A nearly 10‐fold increase in antioxidant activity was observed, rising from 0.58 µmol TE/g in the control to 5.03 µmol TE/g at 30% substitution of both fat and wheat flour. Total phenolic content and antioxidant activity performed parallel trends in the cookie samples, both increasing with higher substitution levels (Figure 4). This observation is consistent with the findings of Dadalı et al. (2025), who reported similar increases in gluten‐free cookies as the proportion of artichoke bracts used as a rice flour substitute increased. In another study, the use of artichoke byproducts to enrich breadsticks was evaluated, and it was concluded that as the incorporation rate of artichoke byproducts increased compared to the control, both total phenolic compounds and antioxidant activity also increased (Cannas et al. 2024). In their study, Ammar et al. (2024) found that using 15% artichoke bracts as a wheat flour substitute in a cookie formulation increased the total phenolic compounds from 0.01 mg GAE/g to 1.75 mg GAE/g. Similarly, another study determined that biscuits enriched with artichoke fiber showed higher antioxidant activity compared to the control sample and the sample produced with pea fiber, referred to as the reference (San José et al. 2023). In a study by Cansev et al. (2024), it was found that the total phenolic compounds and antioxidant capacity of cookies enriched with artichoke byproducts increased with increasing amounts of artichoke flour. The results obtained in the present study, similar to studies with artichoke waste, showed that the addition of artichoke bracts resulted in an increase in total phenolic compounds and antioxidant capacity.

### 3.6 Effect of Using Artichoke Bracts on Sensory Properties of Cookies

**FIGURE 5:**
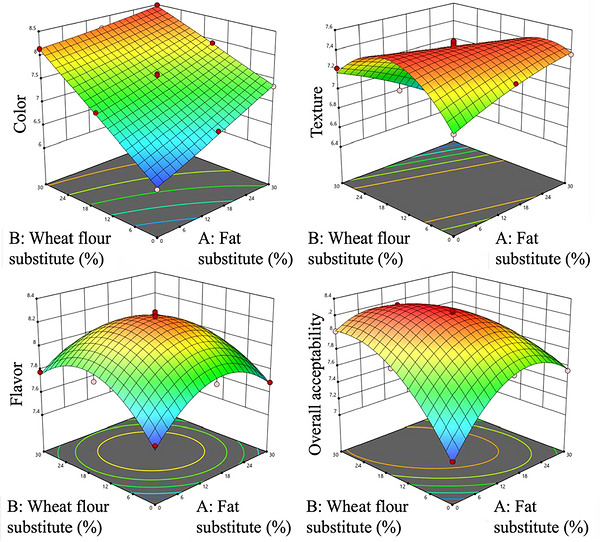
The calculated effect of fat and wheat flour substitution on the sensory properties of cookies.

Color, as a key sensory attribute of cookies, was linearly affected by the substitution levels of fat and wheat flour (*p* < 0.05) (Table 2). Regarding texture, fat substitution showed a significant linear effect, while wheat flour substitution exhibited both linear and quadratic effects (*p* < 0.05). Flavor was quadratically affected by fat and wheat flour substitution (*p* < 0.05), while overall acceptability exhibited both linear and quadratic responses to these variables (*p* < 0.05). In addition, the interaction between the two substitution factors significantly influenced all sensory attributes (color, texture, flavor, and overall acceptability) (*p* < 0.05). Color scores increased with higher levels of artichoke bract substitution (Figure 5), ranging from 6.15 in the control to 8.49 at 30% substitution of both fat and wheat flour. Positive changes in texture were observed as the substitution ratio of fat and wheat flour in the cookies was increased from 0% to 15%. The highest texture score (7.49) was found in the sample with 15% fat substitute and 15% wheat flour substitute (Exp. 3). However, when the substitution ratio was increased from 15% to 30%, lower texture scores were observed. The lowest texture score (6.52) was found in the sample with 30% fat substitute and 30% wheat flour substitute. It was observed that adding 15% artichoke bracts to the cookie formulation increased the flavor and overall acceptability scores compared to the control. Cookies containing 15% fat and 15% wheat flour substitutes were found to exhibit higher flavor intensity and overall acceptability compared to cookies without artichoke bracts.

### 3.7 Optimization and Confirmation

The numerical optimization of fat and wheat flour substitution ratios was performed using the desirability function approach. As a result of the optimization, the optimum fat substitution level was determined as 14.26%, while the optimum wheat flour substitution level was 23.74%. In order to evaluate the agreement between the model‐predicted values and the experimental data, five confirmation experiments were conducted using the cookie formulation having the optimum levels of fat and wheat flour substitution. The results of the confirmation experiments are presented in Table 4. According to the confirmation results, no statistically significant difference was observed between the model‐predicted and experimental values (*p* > 0.05).

**TABLE 4:** Model confirmation results of responses.

Responses	Predicted result	Experimental result^a^	Mean standard error	Difference	Error (%)^b^
Moisture (g/100 g)	12.90	13.03 ± 0.15	0.09	0.21	0.95
Baking loss (%)	11.95	11.72 ± 0.15	0.10	−0.26	1.97
Spread ratio	1.71	1.66 ± 0.04	0.02	−0.04	2.98
Hardness (N)	43.44	43.16 ± 0.95	0.50	0.49	0.64
Fracturability (mm)	52.36	51.98 ± 0.31	0.18	0.56	0.73
L*	57.65	58.16 ± 0.60	0.38	0.51	0.89
a*	3.71	3.63 ± 0.20	0.13	−0.09	2.10
b*	23.01	23.32 ± 0.55	0.34	0.35	1.35
Total phenolic compounds (mg GAE/g)	1.02	1.07 ± 0.04	0.03	0.06	4.87
Antioxidant activity (µmol TE/g)	3.31	3.22 ± 0.11	0.07	0.08	2.79
Color	7.95	8.04 ± 0.08	0.05	0.09	1.12
Texture	7.22	7.16 ± 0.05	0.03	−0.12	0.83
Flavor	8.15	8.21 ± 0.05	0.03	−0.11	0.76
Overall acceptability	8.24	8.19 ± 0.03	0.03	0.05	0.69

^a^ The analysis results were given as arithmetic mean ± standard deviation.

^b^ Error (%) = (|*y*_exp_ − *y*_pre_|/*y*_exp_) × 100.

### 3.8 Comparison of Optimum Cookie and Control Cookie

The comparative analysis of the control and optimized cookies is presented in Table 5. The optimized formulation showed significantly higher moisture content compared to the control (*p* < 0.05), which can be explained by the high water‐binding capacity of fiber‐rich artichoke bracts, enhancing moisture retention in the cookie structure (Boubaker et al. 2016). The ash content of the control cookie was 0.82 g/100 g, whereas it increased significantly to 1.88 g/100 g in the optimized cookie (*p* < 0.05). A comparable effect was reported by Melini et al. (2025), where the inclusion of 10% artichoke bracts in rusk yielded an ash content of 2.67 g/100 g. However, protein content did not differ significantly between control and optimized samples (*p* > 0.05). In contrast, total dietary fiber content increased significantly with artichoke bract incorporation (*p* < 0.05), reflecting their richness in both soluble and insoluble fiber fractions (Ayuso et al. 2024). According to Regulation (EC) No 1924/2006 of the European Parliament and of the Council (European Commission 2006), foods containing at least 6 g of dietary fiber per 100 g may be classified as “high fiber.” Accordingly, the optimized cookie formulation, with a dietary fiber content of 10.14 g/100 g, met the criteria for high‐fiber products. Consistent with these findings, Bavaro, De Bellis, Montemurro, et al. (2025) also reported the production of high‐fiber bread enriched with artichoke bracts.

**TABLE 5:** Comparison of optimum cookie and control cookie.

	Control cookie	Optimum cookie
Moisture (g/100 g)	9.40 ± 0.15^a^	13.25 ± 0.21^b^
Ash (g/100 g)	0.82 ± 0.12^a^	1.88 ± 0.02^b^
Fat (g/100 g)	26.96 ± 0.43^b^	22.65 ± 0.14^a^
Protein (g/100 g)	7.28 ± 0.03^a^	7.40 ± 0.03^a^
Total dietary fiber (g/100 g)	1.73 ± 0.04^a^	10.14 ± 0.11^b^

*Note*: The analysis results are expressed as mean ± standard deviation. Values in the same row with identical superscripts do not differ significantly (*p* > 0.05).

## 4 Conclusion

Artichoke bracts were shown to be a viable partial substitute for fat and wheat flour in cookie formulations. The application of RSM enabled successful optimization of the formulation, resulting in an optimum composition of 14.26% fat substitute and 23.74% wheat flour substitute using artichoke bracts. The optimized cookies showed improved nutritional and functional properties compared to the control sample. In particular, increases in moisture content, ash content, total dietary fiber, hardness, fracturability, total phenolic compounds, and antioxidant activity were observed. In contrast, fat content, baking loss, spread ratio, and color values (L*, a*, b*) decreased, indicating structural and compositional modifications induced by the incorporation of artichoke bracts. From a nutritional perspective, the optimized formulation can be classified as a high‐fiber product, highlighting its potential contribution to the development of healthier bakery products. Sensory evaluation results showed that the optimized cookie exhibited higher consumer acceptability than the control, indicating improved sensory quality with the incorporation of artichoke bracts. Overall, the findings highlight the potential of artichoke bracts as both a functional ingredient and a sustainable solution for food waste valorization. Their incorporation into cookie formulations enhanced nutritional quality without compromising technological and sensory properties, supporting the development of value‐added bakery products within a sustainable food production framework.

## Author Contributions

**Ceyda Dadalı**: conceptualization, investigation, methodology, formal analysis, supervision, writing – original draft, writing – review and editing, data curation. **Yağmur Özcan**: data curation, formal analysis, writing – review and editing, writing – original draft, investigation.

## Conflicts of Interest

The authors declare no conflicts of interest.
